# Supplementary material for: Mapping of shore area wetlands in Lake Tana Biosphere Reserve, Northwest Ethiopia using Sentinel-1A SAR and multi-source data
Source: PLoS One. 2025 Oct 16;20(10):e0317391. doi: 10.1371/journal.pone.0317391 (PMC12530554; doi:10.1371/journal.pone.0317391)
Supplement: S1 Table — (DOCX) [file pone.0317391.s001.docx]

| **Data Type (Data Input)** | **Source** | **Data Output** | **Specifications**  **/Resolution** | **Date** |
| --- | --- | --- | --- | --- |
| Digital Elevation Model (DEM) | National Aeronautics and Space Administration(NASA) Earth Observatory (public domain): http://earthobservatory.nasa.gov/ | Topographic  Elevation in meter above sea level (MASL) utilized to delineate the boundaries of the wetlands from the land (terrestrial)-ward side | 15m resolution | 2021 |
| Lake Tana Bathymetry | Kebedew MG, Tilahun SA, Zimale FA, Belete MA, Wosenie MD, Steenhuis TS. Relating Lake Circulation Patterns to Sediment, Nutrient, and Water Hyacinth Distribution in a Shallow Tropical Highland Lake. Hydrology. 2023;10(9) | The first category (0-2 meters depth) extracted from the bathymetry map to define the boundaries of shoreline wetlands from the lake-ward side | 4681 Lake Depth three dimensional Point Data (X,Y, Depth) | 2017 |
| Major Soil Types Lake Tana Sub-basin | Amhara Design and Supervision Works Enterprise(ADSWE) | Hydric and Non-hydric soil types | Projection: UTM,  Spheroid: WGS 1984,Zone:37 and 1:20,000 scale soil survey | 2015 |
| Wetland Field Inventory and Observation on hydrophytic vegetation survey, hydric soil field observation, wetland hydrology field observation, and elevation ground truth using a handheld GARMIN GPSmap 62s | Wetland field survey | Ground Truth Point Data | Number of Ground Truth points | May 2021 and October 2021 wetland field survey |
| Sentinel-1A SAR | Copernicus Open Access Hub (<https://scihub.copernicus.eu/>). | Hydrophytic/Non-hydrophytic Vegetation and Wetland Hydrology | 10m resolution | May 2021 and Nov 2021 |
