# Supplementary material for: Mapping of shore area wetlands in Lake Tana Biosphere Reserve, Northwest Ethiopia using Sentinel-1A SAR and multi-source data
Source: PLoS One. 2025 Oct 16;20(10):e0317391. doi: 10.1371/journal.pone.0317391 (PMC12530554; doi:10.1371/journal.pone.0317391)
Supplement: S2 Table — (DOCX) [file pone.0317391.s002.docx]

| **No** | **Class Name** | **Training polygon count** | **Accuracy polygon count** |
| --- | --- | --- | --- |
| 1. | Forest | 198 | 87 |
| 2. | Built up | 336 | 190 |
| 3. | Invasive of water hyacinth | 209 | 143 |
| 4. | Cultivated land | 461 | 443 |
| 5. | Hydrophic vegetation | 603 | 584 |
| 6. | Plantation of Eucalyptus trees | 64 | 68 |
| 7. | Shrub land | 110 | 100 |
| 8. | Water body | 2747 | 1561 |
| 9. | Total Polygons | 4,728 | 3,176 |
