# Supplementary material for: Mapping of shore area wetlands in Lake Tana Biosphere Reserve, Northwest Ethiopia using Sentinel-1A SAR and multi-source data
Source: PLoS One. 2025 Oct 16;20(10):e0317391. doi: 10.1371/journal.pone.0317391 (PMC12530554; doi:10.1371/journal.pone.0317391)
Supplement: S3 Table — HS refers to Hydric Soil; HV refers to Hydrophytic Vegetation; WH refers to Wetland Hydrology; and TP refers to Topographic Position. (DOCX) [file pone.0317391.s003.docx]

| **Object ID** | **Layer Name in Raster** | **HS Layer** | **HV Layer** | **WH Layer** | **TP Layer** |
| --- | --- | --- | --- | --- | --- |
| 1 | **HS Layer** | 1 | 0.33 | 0.25 | 3 |
| 2 | **HV Layer** | 3 | 1 | 0.5 | 4 |
| 3 | **WH Layer** | 4 | 3 | 1 | 5 |
| 4 | **TP Layer** | 0.33 | 0.25 | 0.2 | 1 |
