# Supplementary material for: Mapping of shore area wetlands in Lake Tana Biosphere Reserve, Northwest Ethiopia using Sentinel-1A SAR and multi-source data
Source: PLoS One. 2025 Oct 16;20(10):e0317391. doi: 10.1371/journal.pone.0317391 (PMC12530554; doi:10.1371/journal.pone.0317391)
Supplement: S4 Table — (DOCX) [file pone.0317391.s004.docx]

| **Object**  **ID** | **Layer Name**  **in Raster** | **HS**  **Layer** | **HV**  **Layer** | **WH**  **Layer** | **TP**  **Layer** | **CI** | **RI** | **CR** | **Notes** |
| --- | --- | --- | --- | --- | --- | --- | --- | --- | --- |
| 1 | **HS Layer** | 1 | 0.33 | 0.25 | 3 | 0.086 | 0.89 | 0.096 | Consistent enough |
| 2 | **HV Layer** | 3 | 1 | 0.5 | 4 | 0.086 | 0.89 | 0.096 | Consistent enough |
| 3 | **WH Layer** | 4 | 3 | 1 | 5 | 0.086 | 0.89 | 0.096 | Consistent enough |
| 4 | **TP** | 0.33 | 0.25 | 0.2 | 1 | 0.086 | 0.89 | 0.096 | Consistent enough |
