# Supplementary material for: Mapping of shore area wetlands in Lake Tana Biosphere Reserve, Northwest Ethiopia using Sentinel-1A SAR and multi-source data
Source: PLoS One. 2025 Oct 16;20(10):e0317391. doi: 10.1371/journal.pone.0317391 (PMC12530554; doi:10.1371/journal.pone.0317391)
Supplement: S5 Table — (DOCX) [file pone.0317391.s005.docx]

| **FID** | **Shape *** | **Depth Interval** | **Area(ha)** |
| --- | --- | --- | --- |
| 0 | Polygon | 0-2m | 24890.54 |
| 1 | Polygon | 2-4m | 7379.55 |
| 2 | Polygon | 4-6m | 14905.12 |
| 3 | Polygon | 6-8m | 23427.26 |
| 4 | Polygon | 8-10m | 38589.23 |
| 5 | Polygon | 10-12m | 57485.92 |
| 6 | Polygon | 12-13m | 55692.06 |
| 7 | Polygon | 13-14.92m | 83149.89 |
