# Supplementary material for: Mapping of shore area wetlands in Lake Tana Biosphere Reserve, Northwest Ethiopia using Sentinel-1A SAR and multi-source data
Source: PLoS One. 2025 Oct 16;20(10):e0317391. doi: 10.1371/journal.pone.0317391 (PMC12530554; doi:10.1371/journal.pone.0317391)
Supplement: S6 Table — (DOCX) [file pone.0317391.s006.docx]

| **Major Soil Type** | **Hydric Nature** | **Sub-classification** | **Area in hectare** |
| --- | --- | --- | --- |
| Acrisols | Non-hydric | Non-hydric | 156.19 |
| Alisols | Non-hydric | Non-hydric | 6105.11 |
| Cambisols | Non-hydric | Non-hydric | 4524.73 |
| Ferralsols | Non-hydric | Non-hydric | 1016.11 |
| Fluvisols | Hydric | Permanently Hydric | 9760.32 |
| Gleysols | Hydric | Permanently Hydric | 8265.97 |
| Lake Tana | Hydric | Water Body | 303260.16 |
| Leptosols | Non-hydric | Non-hydric | 7708.54 |
| Lixisols | Non-hydric | Non-hydric | 56.75 |
| Luvisols | Non-hydric | Non-hydric | 8693.89 |
| Nitisols | Non-hydric | Non-hydric | 8725.15 |
| Regosols | Non-hydric | Non-hydric | 957.38 |
| Towns | Non-hydric | Non-hydric | 5814.73 |
| Vertisols | Hydric | Seasonally Hydric | 34924.07 |
| Gleysols | Hydric | Permanently Hydric | 2200.41 |
| Total Hydric Soil Cover |  |  | 55150.78 |
