# Supplementary material for: Mapping of shore area wetlands in Lake Tana Biosphere Reserve, Northwest Ethiopia using Sentinel-1A SAR and multi-source data
Source: PLoS One. 2025 Oct 16;20(10):e0317391. doi: 10.1371/journal.pone.0317391 (PMC12530554; doi:10.1371/journal.pone.0317391)
Supplement: S7 Table — (DOCX) [file pone.0317391.s007.docx]

| **Classified Class** | **Shrub land** | **Water Body** | **Built-up** | **Hydrophic** | **Plantation** | **Cultivated**  **land** | **Forest** | **Total**  **(User)** |
| --- | --- | --- | --- | --- | --- | --- | --- | --- |
| Shrub land | 13 | 0 | 3 | 13 | 0 | 0 | 3 | 32 |
| Water Body | 0 | 533 | 0 | 9 | 0 | 0 | 0 | 542 |
| Built-up | 2 | 1 | 5 | 10 | 1 | 0 | 2 | 21 |
| Hydrophic | 64 | 5 | 79 | 356 | 19 | 82 | 22 | 627 |
| Plantation | 0 | 0 | 0 | 0 | 0 | 0 | 0 | 0 |
| Cultivated land | 4 | 0 | 10 | 18 | 0 | 27 | 0 | 59 |
| Forest | 3 | 0 | 0 | 3 | 0 | 0 | 0 | 6 |
| Unknown | 0 | 0 | 0 | 0 | 0 | 0 | 0 | 0 |
| Total (Producers) | 86 | 539 | 97 | 409 | 20 | 109 | 27 | 1287 |
