# Supplementary material for: Mapping of shore area wetlands in Lake Tana Biosphere Reserve, Northwest Ethiopia using Sentinel-1A SAR and multi-source data
Source: PLoS One. 2025 Oct 16;20(10):e0317391. doi: 10.1371/journal.pone.0317391 (PMC12530554; doi:10.1371/journal.pone.0317391)
Supplement: S8 Table — (DOCX) [file pone.0317391.s008.docx]

| **No** | **Major land use/cover** | **Area(ha)** |
| --- | --- | --- |
| 1. | Built-up | 3505.47 |
| 2. | Cultivated land | 18939.38 |
| 3. | Floodplain | 23432.39 |
| 4. | Forest | 3209.08 |
| 5. | Hydrophic | 72782.43 |
| 6. | Invasive of WH | 1989.43 |
| 7. | Plantation of Eucalyptus | 30.28 |
| 8. | Scrubland | 8434.68 |
| 9. | Water Bodies | 285987.54 |
