# Supplementary material for: Mapping of shore area wetlands in Lake Tana Biosphere Reserve, Northwest Ethiopia using Sentinel-1A SAR and multi-source data
Source: PLoS One. 2025 Oct 16;20(10):e0317391. doi: 10.1371/journal.pone.0317391 (PMC12530554; doi:10.1371/journal.pone.0317391)
Supplement: Attachment — Submitted filename: Response to Reviewers.docx [file pone.0317391.s010.docx]

**Response to Reviewers**

**PONE-D-24-59965**

**Mapping of Shore Area Wetlands in Lake Tana Biosphere Reserve, Ethiopia Using Sentinel-1A SAR and Multi-Source Data**

| **No.** | **Academic Editor and Reviewers-Comments** | **Response to Academic Editor and Reviewers** |
| --- | --- | --- |
|  | **Acknowledgements** | The authors wish to thank the academic editor and anonymous reviewers for their valuable comments and recommendations on the original version of this study. |
|  | **Academic Editor Comments** | **Response to Academic Editor** |
| **1.** | Please ensure that your manuscript meets PLOS ONE's style requirements, including those for file naming | **Now our manuscript meets** PLOS ONE's style requirements by strictly following the PLOS ONE style templates. Figure and Table Captions based on the template. Figure files saved as “Fig1.tif”. File Naming for Supporting Information based on the template and supporting Information files saved as “S1_Fig.tif”, |
| **2.** | We note that your Data Availability Statement is currently as follows: All relevant data are within the manuscript and its supporting information files. For example, authors should submit the following data: (a) The values behind the means, standard deviations and other measures reported; (b) The values used to build graphs; and (c) The points extracted from images for analysis. | Thank you for your comments. Supporting tables of **S1Table.** Table Summary of data types and sources used in the study; **S2 Table*.*** Description of Training and Accuracy Counts for this study; **S3 Table.** AHP matrix produced by AHP (step1) script and filled by the user; **S4 Table*.*** AHP matrix produced by AHP (step2) script that generate weight, consistency index (CI), the average consistency index (RI), consistency ratio (CR) and Notes; **S5 Table.** Results of Lake Tana's bathymetry were interpolated using the Inverse Distance Weighting (IDW) method; **S6 Table.** Major Soil types, hydric nature, sub-classes and their area coverage in hectare; **S7 Table.** Confusion Matrix; and **S8 Table.** Major land use/cover and their area coverage in hectare are included under supplementary data. |
| **3.** | Please amend your list of authors on the manuscript to ensure that each author is linked to an affiliation. Authors’ affiliations should reflect the institution where the work was done (if authors moved subsequently, you can also list the new affiliation stating “current affiliation:….” as necessary) | List of authors on the manuscript amended as follow:  **Yirga Kebede Wondim****<https://orcid.org/0000-0002-9016-6105> ^1,*^, Ayalew Wondie Melese****<https://orcid.org/0000-0002-9016-6105> ^1^ and Workiyie Worie Assefa****<https://orcid.org/0000-0002-9016-6105> ^2^**  ^1^Department of Aquatic and Wetland Ecosystem Management, School of Fisheries and Wildlife, College of Agriculture and Environmental Sciences, Bahir Dar University, P.O. Box 79, Bahir Dar, Ethiopia.  ^2^Department of Biology and Blue Nile Water Institute, College of Science, Bahir Dar University, P.O. Box 79, Bahir Dar, Ethiopia.  ^*^ Corresponding author |
| **4.** | We note that Figures 1, 3, 4, 6, 7, 8, 9, in your submission contain [map/satellite] images, which may be copyrighted. All PLOS content is published under the Creative Commons Attribution License (CC BY 4.0), which means that the manuscript, images, and Supporting Information files will be freely available online, and any third party is permitted to access, download, copy, distribute, and use these materials in any way, even commercially, with proper attribution. For these reasons, we cannot publish previously copyrighted maps or satellite images created using proprietary data, such as Google software (Google Maps, Street View, and Earth). For more information, see our copyright guidelines: http:// journals.plos.org/plose one/s/licenses-and-copyright.  We require you to either (1) present written permission from the copyright holder to publish these figures specifically under the CC BY 4.0 license, or (2) remove the figures from your submission:  a.You may seek permission from the original copyright holder of Figures 1, 3, 4, 6, 7, 8, 9, to publish the content specifically under the CC BY 4.0 license.  We recommend that you contact the original copyright holder with the Content Permission Form (http://journals.plos.org/plosone/s/file?id=7c09/content-permission-form.pdf) and the following text: “I request permission for the open-access journal PLOS  ONE to publish XXX under the Creative Commons Attribution License (CCAL) CC BY 4.0 (http://creativecommons.org/licenses/by/4.0/). Please be aware that this license allows unrestricted use and distribution, even commercially, by third parties. Please reply and provide explicit written permission to publish XXX under a CC BY license and complete the attached form.” | - Figure 1. Location map of the study area (Attribution: Esri, Delorme, HERE, Tom Tom, Intermap, Increment P Corp, GEBCO, USGS, FAO, NPS, NRCAN, GeoBase, IGN, Kadaster NL, Ordance Survey, Esri Japan, METI, Esri China(Hong Kong), swisstopo, MapmyIndia, and the GIS User Community). - Figure 3 Topographic position-mapping processes. We contacted the original copyright holder with the Content Permission Form. We get permission from the original copyright holder to use bathymetry map Lake Tana: Link to the Content: https://onlinelibrary.wiley.com/doi/abs/10.1002/esp.5118 and we uploaded the completed Content Permission Form as an "Other" file with our revised manuscript submission. - Figure 4 Hydric soil mapping processes: Previously we adopted a reference soil group map of Ethiopia [Ali A, Erkossa T, Gudeta K, Abera W, Mesfin E, Mekete T, et al. Reference soil groups map of Ethiopia based on legacy data and machine learning-technique: EthioSoilGrids 1.0. Soil. 2024; 10(1):189–209.] , based on legacy data and machine learning techniques (EthioSoilGrids 1.0), which is the first of its kind for Ethiopia. However, after comments related to copyright issue received from the academic editor, we excluded from our work. We used only field observation data along with a digital format of the Tana sub-basin soil survey, unpublished project work. - Figure 6*.* The difference in backscattering between the Gulf of Lake Tana region covered by water and other types of land cover. This is the images of Sentinel-1 SAR GRD data freely downloading from European Space Agency and 3km buffer produced in our study using Arc GIS software. We acknowledge European Space Agency for providing access to Sentinel-1 Data product that were instrumental in advancing this research at the Acknowledgment section. - Figure 7 Shore area wetland indicator maps. We adapted and improved. The copy right issues already addressed at Figure 3,4 &6. - Figure 8 Shore area wetland in Lake Tana: Shape files of Fogera and Dembia floodplains for illustrative purposes only. We specified in the figure caption text Shape files of Fogera and Dembia floodplains illustrative purposes only. Area coverage of Fogera and Dembia floodplains in hectares removed from the text. - Figure 9 Shore area wetland groups in Lake Tana removed the manuscript |
|  | Please upload the completed Content Permission Form or other proof of granted permissions as an "Other" file with your submission. | We uploaded the Completed Content Permission Form in the file name: Wondim_Copyright as an "Other" file with our submission of revised manuscript. |
|  | In the figure caption of the copyrighted figure, please include the following text: “Reprinted from [ref] under a CC BY license, with permission from [name of publisher], original copyright [original copyright year].” | Fig 3 Topographic position mapping processes. Lake Tana Bathymetry remapped from [35] under a CC BY license, with permission from [John Wiley & Sons, Ltd.], original copyright [© 2021}. |
|  | b. If you are unable to obtain permission from the original copyright holder to publish these figures under the CC BY 4.0 license or if the copyright holder’s requirements are incompatible with the CC BY 4.0 license, please either i) remove the figure or ii) supply a replacement figure that complies with the CC BY 4.0 license. Please check copyright information on all replacement figures and update the figure caption with source information. If applicable, please specify in the figure caption text when a figure is similar but not identical to the original image and is therefore for illustrative purposes only.  The following resources for replacing copyrighted map figures may be helpful:  USGS National Map Viewer (public domain): http://viewer.nationalmap.gov/viewer/  The Gateway to Astronaut Photography of Earth (public domain): http://eol.jsc.nasa.gov/sseop/clickmap/  Maps at the CIA (public domain): https://www.cia.gov/library/publications/the-world-factbook/index.html and https://www.cia.gov/library/publications/cia-maps-publications/index.html  NASA Earth Observatory (public domain): http://earthobservatory.nasa.gov/  Landsat: http://landsat.visibleearth.nasa.gov/  USGS EROS (Earth Resources Observatory and Science (EROS) Center) (public | .Figure 8 Shore area wetland in Lake Tana:  The copyright map replaced by copyright free resources suggested by the Academic Editor. Shape files of Fogera and Dembia floodplains removed from the map and area coverage of these floodplains included in the manuscript also removed.  Figure 1. Location map of the study area (Attribution: Esri, Delorme, HERE, Tom Tom, Intermap, Increment P Corp, GEBCO, USGS, FAO, NPS, NRCAN, GeoBase, IGN, Kadaster NL, Ordance Survey, Esri Japan, METI, Esri China(Hong Kong), swisstopo, MapmyIndia, and the GIS User Community). |
| 5. | We note you have included a table to which you do not refer in the text of your manuscript. Please ensure that you refer to Table 4 in your text; if accepted, production will need this reference to link the reader to the Table. | All figures, tables in the manuscript and tables included under the supplementary data referred in the text of our manuscript. |
| 6. | Please include a new copy of Table 1 and 2 in your manuscript; the current table is difficult to read. | Tables 1 and 2 in screenshots converted into text. |
|  | **Reviewer-1 Comments** | **Response to Reviewer-1** |
| **1.** | **General Comments:**  The manuscript attempts to address an important challenge in delineating shoreline wetlands by integrating Sentinel-1 SAR data and multi-source indicators (topography, hydric soil, hydrophytic vegetation, and hydrology) to map Lake Tana’s wetlands. While the topic is relevant and the multi-indicator approach is logical, the manuscript suffers from methodological ambiguities and the current quality of the figures significantly detracts from the readability. | Thank you for your critical comments.  Clear methodologies designed. Now the quality and readability of the figures improved. |
| 2. | **Methodology Section Comments:** Some parts of the methodology are unclear and lack sound reasoning. | Additional subsection for further clarification of the methodology included. The following additional subsection for further clarification of the methodology was included:   - Data types and sources - Preprocessing - Segmentation - Attribute calculation - Training Sites Editing - Classification accuracy assessments - Wetland hydrology mapping using SAR data (additional clarification included) |
|  | Lines 142-145. The choice of a 3 km buffer zone and a critical elevation of 1790m requires detailed explanation. | During the pre-field visit of 2021, the maximum distance of the extended shore area wetland from the Lake Tana shoreline during maximum water level is about 3-kilometer and has 1790 meters above sea level. Thus, based on the maximum distance of the extended shore area wetland, we determined our study area to be a 3 km buffer zone around the lake. Besides, the elevation meter above sea level up to 1790 is considered as potential shore area wetland around Lake Tana, where wetlands receive water from an adjacent large freshwater lake, Tana. |
|  | Mapping Hydrophytic Vegetation Using SAR Data. In this section, the number and date of Sentinel-1 images used are unknown. | A total of 10 Sentinel-1 GRD images in the Interferometric Wide swath (IW) mode (10m resolution) with VV and VH polarizations of May 2021 and Nov 2021. |
|  | The construction of training datasets is unclear. How to generate training site polygons from ground truth points? | Thank you for your critical comments, dear reviewer. We clarified the steps for generating training site polygons from ground truth points by including the Training Sites Editing Subsection.  **Training Sites Editing**  Before we perform a classification or an accuracy assessment, we must have ground-truth data. In Object Analyst of PCI Geomatics, we could collect training samples for both the supervised classification and accuracy assessment in the same window of Training Sites Editing [42]. In the Training Sites Editing wizard, the sample type includes both training and accuracy assessment. Both training and accuracy polygons could be created by both manual edit and automatic import. In this study, we used manual or on-screen interpretation in the viewer and selected segments of various classes by selecting Create Polygon, Assign, Unassign, and Reset Class. Our ground truth shape files did not import automatically; rather, we used them for overlay purposes to create accurate training and accuracy sites. The data for accuracy should be of higher quality than the data used for training or classification [42,43].Thus, creating polygons selected for both training and accuracy assessment was carried out by applying GIS layer information created using the ground truth points gathered through field visits during May 2021 and October 2021 and SAS Planet imagery, resulting in these ground truth shape files overlaid with Sentinel-1A SAR images. Using these inputs, separate training and accuracy counts of polygons were created for both training and accuracy assessment using manual editing (S2 Table).  A total of 4,728 training polygons representative of sites including forest (198), built-up areas (336), invasive water hyacinth (209), cultivated land (461), hydrophytic vegetation (603), eucalyptus tree plantations (64), shrub land (110), and water bodies (2747) were created. Similarly, a total of 3,176 accuracy polygons representative of sites including forest (87), built-up areas (190), invasive water hyacinth (143), cultivated land (443), hydrophytic vegetation (584), eucalyptus tree plantations (68), shrub land (100), and water bodies (1,561) were created. The number of polygons for training and accuracy by individual classes is shown in **S2 Table.** |
|  | How to determine the classification categories? | According to the criteria of wetland classification in the wetland convention and the current situation of land use/cover of shore area wetlands in Lake Tana surveyed during ground truth data collection, we classified the study area into nine land cover types: forest, built-up areas, invasive water hyacinth, cultivated land, hydrophytic vegetation, eucalyptus tree plantations, shrub land, and water bodies. |
|  | Since the discussion section mentioned that the random forest is considered the most effective machine-learning approach for wetland mapping, why is only SVM used here? | The SVM classifier method was chosen in this study for the following reasons: (a) SVM is reported to perform better than Random Forest Trees (RFT) classifier when the training set is small or unbalanced. (b) When two classes are not discriminable linearly in a two-dimensional space, the kernel, a mathematical function used by the SVM classifier could separable in a higher-dimensional space (hyperplanes). From four basic kernels, the radial basis function (RBF) kernel provides the best results. With SVM, the objective is to find the optimal separating hyperplane (decision surface, boundaries) by maximizing the margin between classes, which is achieved by analyzing the training samples located at the edge of the potential class. |
|  | Wetland Hydrology Mapping Using SAR Data. Since the hydrophytic vegetation mapping section already includes the waterbody category, why do we need another map in this section to extract waterbodies? Again, it is unclear how to use Sentinel-1 images in this section. | In the case of Wetland Hydrology Mapping Using SAR data from Sentinel-1 SAR GRD, features with higher backscatter appear brighter (high backscattering value ranges in decibels (dB)), and water beneath the vegetation in wetland ecosystems where radar pulses are backscattered twice, "double bounce" phenomenon, either from the water’s surface to the vegetation or vice versa, appears semi-darker (medium backscattering value ranges in dB), while those with lower backscatter (low backscattering value ranges in dB) appear darker. Whereas during hydrophytic vegetation mapping, water beneath the vegetation in wetland ecosystems was included under the vegetation cover or grass. In this case, vegetation cover beneath the water, which is in reality wetland cover, may be considered as grassland or even cropland during the growing season. |
|  | Current wetland mapping methods are well- established. A dedicated section comparing this approach with prior methods would strengthen the case for novelty. | Thank you very much for you comment. As per your comments, we included at the end of the introduction, discussion and conclusion section about comparing our combined or multiple approach with the prior methods.  **Introduction section:** We expect that SAR remote sensing data alone could not produce satisfactory wetland classification accuracy; rather, mapping wetlands using multiple wetland indicator approach ensures a more accurate and comprehensive representation of wetlands.  **Discussion section:** Our findings align with prior studies indicating that multi-temporal SAR data enhances wetland classification [40,44,45].While SAR-based methods are effective, our results suggest that the integration of SAR with multi-source data, such as DEMs, can further improve classification accuracy, as highlighted by [43]. Our findings contrast with [42], who reported poor performance of Sentinel-1A SAR for herbaceous wetland mapping in the Biebrza floodplain (Poland), citing coarse resolution and limitations in detecting small wetland features. They achieved an overall accuracy (OAA) of 65% and a Kappa index (KI) of 0.58 using multi-temporal SAR with VV/VH polarization. In comparison, our results demonstrated higher accuracy (OAA = 78.68%, KS = 0.70). Nonetheless, their findings highlighted the superior performance of TerraSAR-X/TanDEM-X images, especially in fully polarimetric mode.  **Conclusion section:** The finding of this study suggests that SAR data alone could not produce satisfactory wetland classification accuracy. We minimize such limitations by integrating spatial geo-information indicators, such as TP and HSs derived from ArcGIS, with data captured by Sentinel-1A SAR, which focuses on hydrophytic vegetation and wetland hydrology, processed through PCI Geomatica Banff software. Mapping wetlands using multiple wetland indicator approach ensures a more accurate and comprehensive representation of shoreline wetlands. |
| 3. | **Comments on Figures and Tables:**  Although the manuscript is well structured, the figures are terrible and affect readability. The key figure showing hydrophytic vegetation mapping (Figure 5) is even a combination of screenshots of the Geomatica Focus interface. Figure 1. The color of the base-map is close to the color of the legend, which interferes with the reading of key information (Figs 1). Figure 2. The demonstration of the proposed workflow should be improved. The arrows are inconsistent, and some text is obscured. Why is there a “Wetland InSAR” when the mapping method in this manuscript only uses SAR backscattering intensity? Figure 3. The scale and extent of the subplots are inconsistent, making it difficult to compare their differences. In addition, it is hard to understand the overlap of the two layers (1786-90 Elevation meter and 0-2 meter Depth) in subplot e. Figure 7 has similar problems. | Thank you for your critical comments. Now the quality and readability of the figures improved. The figure 5 that shows the process flow for OBIA: General workflow of hydrophytic vegetation mapping using object analyst within Geomatica Focus, improved. Figure 1 also changed. Figure 2 General workflow of mapping shore area wetlands also improved. Figure 3 and Figure 7 improved. Wetland InSAR removed. Both hydrophytic vegetation and the wetland hydrology extracted from the same database SAR Database where as the processes and soft wares are different (please check Figure 2 General workflow of mapping shore area wetlands. |
|  | Tables 1 and 2 should not use screenshots | Tables 1 and 2 in screenshots converted into text and moved to supplementary data (**S3 Table.** AHP matrix produced by AHP (step1) script and filled by the user and **S4 Table*.*** AHP matrix produced by AHP (step2) script that generate weight, consistency index (CI), the average consistency index (RI), consistency ratio (CR) and Notes)**.** |
|  | **Reviewer-2 Comments** | **Response to Reviewer-2** |
| 1. | **General Comments:**  I have gone through the manuscript Mapping of Shore Area Wetlands in Lake Tana Biosphere Reserve, Ethiopia Using Sentinel-1A SAR and Multi-Source Data. The manuscript needs more attention of the author. | We gave more attention for your comments so as to improve the quality of our draft manuscript. |
| 2. | **Language:**  The English language should be revised. There are many grammar errors and writing mistakes. | English language revised. |
| 3. | **Abbreviation:**  It is recommended to fully spell out the in the first use of the term in a manuscript. Once the abbreviation has been introduced, it is appropriate to use it throughout the rest of the manuscript. So, readers will better understand the meaning of the abbreviation and its relevance to the topic at hand. Please revise all abbreviations | Now all abbreviations are revised. Once the abbreviation has been introduced, we use the abbreviation throughout the text of the manuscript. For the following phrases, abbreviation have been defined and once we introduced, we use throughout the text of the manucriptions except sub-headings: Hydrophic vegetation(HV), Hydric soil(HS),Wetland Hydrology (WH) and Topographic Position(TP), Lake Bathymetry(LB), Synthetic Aperture Radar(SAR), Digital Elevation Model(DEM),Object Based Image Analysis(OBIA) and Support Vector Machines (SVMs). |
| 4. | **Introduction Section:**  The introduction section should be supported by recent literature reviews that used remote sensing such as optical and radar data to detect water bodies: It is not the first study used this model in the research region. I think the authors could read them and cite these. please see  • Abou Samra RM, El-Barbary SM (2018) The use of remote sensing indices for detecting environmental changes: a case study of North Sinai, Egypt Spatial Information Research 26:679-689. https://doi.org/10.1007/s41324-018-0211-1  • Abou Samra R.M. (2022). Dynamics of human-induced lakes and their impact on land surface temperature in Toshka Depression, Western Desert, Egypt. Environmental Science and Pollution Research, 29, 20892-20905.  • Abou Samra RM, Ali R, Halder B, & Yaseen ZM (2024) Assessing the Catastrophic Environmental Impacts on Dam Breach Using Remote Sensing and Google Earth Engine. Water Resources Management:1-17 | Thank you for your suggestion. The introduction section supported by recent literature reviews (see paragraph 2: from line 3-5).  In Egypt, remote sensing technology has been utilized to detect environmental changes and land surface temperature [12,13]. To create a thorough grasp of wetland dynamics throughout Africa, the work of [14] integrates carbon stock calculation, fragmentation analysis, and spatial mapping using GIS and remote sensing technologies. |
| 5. | **Methodology Section:**  You should add a section about characteristics of your study area including causes of selection, and the meteorological conditions. | Based on your suggestion the meteorological conditions of Lake Tana sub-basin in relation to the development and survival of wetlands included( see sub-section of **Description of the study area:** paragraph 4**)** as follow:  Wetlands rely on precipitation (rainfall), surface water, and groundwater for their hydrological needs. Because it serves as a major supply of water and affects water levels, flow patterns, and ultimately the ecology of the wetland, rainfall is essential to the development and survival of wetlands. The seasonal variation of rainfall in Lake Tana sub-basin is controlled by the northward and southward movement of the intertropical convergence zone and the mean annual rainfall varies from 1, 200 to 1, 600 mm[27].Summer is the Lake Tana sub-basin's main rainy season, spanning from mid-June to mid – September, with the sub-basin’s climate consisting mainly of tropical highland monsoon [28].Generally, the southern part of the Lake Tana sub-basin is wetter than the western and the northern parts. |
|  | • The methods section should be well written. Please add section of sources and characteristics of your datasets. | Thank you for your critical comment. Now we included subsection of Data types and sources |
|  | Please clarify your processing steps and software used to process your datasets. | Based on comments, we clearly indicated the processing steps in both text and figures. We also clarify the software used to process our datasets both in the text and in the figure that shows the workflow. |
| **6.** | **Conclusion:**   - Please add the implication of the findings for future research | The implication of the findings of this study for future research indicated in the conclusion section as follow:  The implications of these findings are profound for future research. The finding of this study suggests that SAR data alone could not produce satisfactory wetland classification accuracy. We minimize such limitations by integrating spatial geo-information indicators, such as TP and HSs derived from ArcGIS, with data captured by Sentinel-1A SAR, which focuses on hydrophytic vegetation and wetland hydrology, processed through PCI Geomatica Banff software. Mapping wetlands using multiple wetland indicator approach ensures a more accurate and comprehensive representation of shoreline wetlands. For future mapping, detecting changes and restoring shore area wetlands in Lake Tana, the study result provides important information on which wetland indicators (topographic position, hydric soil, hydrophytic vegetation and wetland hydrology) are highly significant indicators for the existence and restoration of wetland resources. The consistency ratio (CR) value of this study confirms that wetland hydrology could be recognized as the most critical variable among the three defining factors of wetlands. Because neither the characteristic substrates nor the typical biota of wetlands can emerge without appropriate hydrologic regimes. This implies that during mapping and restoring wetlands, priority needs to be given for wetland hydrology followed by hydrophytic vegetation.  The implications of these findings are also profound for future specific practical management recommendations. The results of this study provide a valuable baseline for future practical guidance for the sustainable management and conservation of shoreline wetland resources in the Lake Tana Biosphere Reserve. For managing and restoring shore area wetlands and making policy, the following measures are suggested: Shore area wetlands need to be delineated, and their buffer zone needs to be established using as a reference the results of this study. |
|  | - This write-up should address the current limitations in this field of study | Based on your suggestions, Limitations and future works sub-section at the end of the discussion section included.  **Limitations and future works**  The proposed method of integrating Sentinel-1A SAR with multi-source, multi-temporal data shows promise for generating annual and seasonal wetland maps in the Lake Tana Biosphere Reserve. Combining SAR data with spatial wetland indicators, such as hydric soils and topographic position improves classification accuracy and enables more precise monitoring of shoreline wetland dynamics. Although the study demonstrated that four wetland indicators (TP, HS, HV and WH) effectively map the spatial extent of shoreline wetlands in Lake Tana, northwestern Ethiopia, there are some limitations and remaining issues that might be addressed in future work. Factors that limit mapping of shore area wetlands in Lake Tana Biosphere Reserve were: (a) This study limited on lacustrine fringe wetland type’s spatial scale and temporal scale (absence of change detection). From four multiple wetland indicators, hydric soil, hydrophytic vegetation, and wetland hydrology could be applied for all types of wetlands. However, the approach we applied to extract the topographic position indicator is restricted to lacustrine fringe and tidal fringe wetland types. Spatiotemporal change detection is another application of SAR wetland monitoring. The advent of high spatial resolution remote sensing imagery further supports opportunities to apply change detection with object-based change detection (OBCD). OBCD was not included in this study. This wetland change analysis would provide crucial information about drivers of lacustrine wetland changes in Lake Tana and, consequently, would improve better wetland management in the lacustrine wetland changes in Lake Tana. Therefore, changes in lacustrine wetlands in Lake Tana need to be detected using Sentinel-1 SAR data and Geomatica Banff software packages in future research work. (b) The total amount of carbon stored by each wetland could be estimated by multiplying the total area of the wetland with the value of carbon stock per hectare from the literature. Similarly, carbon emission could be estimated by multiplying the total area of the wetland with its corresponding emission/removal factor from the literature. However, this study focused on mapping area coverage and carbon storage as well as carbon emission were not included in this research work. Therefore, using the total area of the lacustrine wetlands in Lake Tana estimated by this study, the carbon storage and emission need to be estimated for two-wetland degradation states (pristine and drained conditions) in future research work. (c) This study depend on secondary data of LB and major soil type, which reveal mapping error. These factors need to be considered in future research. (d) SAR data processing in this study is supported by PCI Geomatica software, whose accessibility is constrained by licensing and copyright restrictions. Besides licensing and copyright restrictions, processing SAR data needs massive computation, which lowers the processing speed using a personal computer. Future research work needs to be done to process SAR data using Google Earth Engine integrated with a large number of free remote sensing images and providing remote computation. |
