# Supplementary material for: Mapping of shore area wetlands in Lake Tana Biosphere Reserve, Northwest Ethiopia using Sentinel-1A SAR and multi-source data
Source: PLoS One. 2025 Oct 16;20(10):e0317391. doi: 10.1371/journal.pone.0317391 (PMC12530554; doi:10.1371/journal.pone.0317391)
Supplement: Attachment — Submitted filename: Response to Reviewers_2.docx [file pone.0317391.s011.docx]

**Response to Reviewers-2**

**PONE-D-24-59965**

**Mapping of Shore Area Wetlands in Lake Tana Biosphere Reserve, Northwest Ethiopia Using Sentinel-1A SAR and Multi-Source Data**

| **No.** | **Academic Editor and Reviewers-Comments** | **Response to Academic Editor and Reviewers** |
| --- | --- | --- |
|  | **Acknowledgements** | The authors wish to thank the academic editor and anonymous reviewers for their valuable comments and recommendations on the second version of the manuscript after considering comments. |
|  | **Academic Editor Comments** | **Response to Academic Editor** |
| **1.** | If the reviewer comments include a recommendation to cite specific previously published works, please review and evaluate these publications to determine whether they are relevant and should be cited. There is no requirement to cite these works unless the editor has indicated otherwise. | Reviewer-2 suggested three recent research works to read and cite this literature. So we reviewed and evaluated these publications. Finally, we decided two of them were found relevant to this study, whereas the last one (Abou Samra RM, Ali R, Halder B, & Yaseen ZM (2024) Assessing the Catastrophic Environmental Impacts on Dam Breach Using Remote Sensing and Google Earth Engine. Water Resources Management (1-17) found them not related and relevant to this study. Thus, we did not include it in this study. |
| **2.** | Please review your reference list to ensure that it is complete and correct. If you have cited papers that have been retracted, please include the rationale for doing so in the manuscript text, or remove these references and replace them with relevant current references. Any changes to the reference list should be mentioned in the rebuttal letter that accompanies your revised manuscript. If you need to cite a retracted article, indicate the article’s retracted status in the References list and also include a citation and full reference for the retraction notice. | Thank you for your comments.  Now we reviewed and updated every reference carefully. We used Mendeley Reference Manager for desktop software. Now we updated every reference manually using the Details tab of Mendeley Reference Manager for Desktop Software by the following steps: (a) Right-click on the article title in the main window and select update details. This will update the details with the latest version of the entry from the Mendeley Web Catalog. (b) Check the type of the reference (journal article, book, book section, report, thesis, etc.).(c) Authors based on Vancouver style. (d) Other details. (e) Click Details are correct at the Details tab. (f) Finally, go to the Word document, click References, click Insert Citation, go to Mendeley, select the reference to be cited, and then click Cite from the Mendeley Desktop. In our manuscript the revised references in our reference list highlighted changes in purple color made to the original version. We also uploaded this as a separate file labeled 'Revised Manuscript with Track Changes.'  We also checked every reference to determine or check if the paper was retracted or not using journal papers from the Retraction Watch Database. The Retraction Watch Database (https://retractiondatabase.org/RetractionSearch.aspx?). required to have criteria entered in at least one of the following fields: Author, Title, Journal, Publisher, Affiliation(s), Country(s), Article Type(s), Original Paper Date Ranges, Retraction or Other Notices Date Ranges, PubMed ID, and DOI for either the original paper, notice, or both. Our search result indicated that No Retractions found matching selected criteria for every reference list, and we did not get any retracted references.  We replaced a few references in the press previously with published versions of the same journal article. |
| **3.** | **Additional Editor Comments:**  The manuscript has been greatly improved and is nearly ready for publication. There are a couple methodological points that need to be clarified before publication. | Methodological issues suggested by **Reviewer #1 clarified.** |
|  | **Reviewer-1 Comments** | **Response to Reviewer-1** |
| **1.** | **Reviewer #1:** The manuscript has greatly improved in terms of clarity and figures. Some minor comments are needed to be addressed before publication. | We appreciate the reviewer's insightful comments and the opportunity to clarify the methods we applied. |
|  | 1.1 Mapping hydrophytic vegetation using SAR data. i. It is unclear how you use SAR images from two different periods (May and October). Do you composite them into a single image? If so, why do you select these two months? | We utilized SAR data from two distinct periods: May and October. These months were intentionally chosen to capture the seasonal dynamics of the hydrology and vegetation within the study area.  See Data types and sources sub-section-paragraph-2:  May 2021, the end of the peak dry season, and Nov 2021, the end of the peak wet season, were downloaded and processed to produce a hydrophytic vegetation indicator map and a wetland hydrology indicator map (S1Table).May marks the transition from the dry season to the onset of the wet season in our region. During this time, temporary wetlands and seasonal floodplains are typically at their minimum extent, and vegetation is less vigorous. SAR data from this period is instrumental in delineating permanent water bodies and areas of persistent inundation. October coincides with the peak of the wet season, when seasonal wetlands and floodplains reach their maximum extent. Elevated soil moisture and inundation levels, coupled with vigorous growth of hydrophytic vegetation, may result in distinct backscatter signatures. As a result, we employed a temporal analysis approach, or the seasonal-rule-based wetland classification method, to generate an annual wetland map focusing on the backscatter coefficient from both time periods to leverage the seasonal changes in land surface characteristics.  Please see sub-section Hydrophytic and non-hydrophytic vegetation classification using SVM-paragraph-1:  We employed a temporal analysis approach, or the seasonal wetland classification method, to generate an annual hydrophytic wetland indicator map. Dry season, May 2021 image used because dry season images seem to have higher accuracy than the wet season images to successfully determine permanent shore area wetlands hydrophytic vegetation cover from the other land cover categories. However, seasonally flooded shore area wetlands hydrophytic vegetation classified as other covers mainly grasslands, and the map removed flooded wetlands and seasonal marshes. Therefore, we also considered the wet season, the October 2021 image that could include seasonal shore area wetlands hydrophytic cover. Finally, we updated the dry season map using Arc GIS software: Analysis Tools: Overlay: Update. We exported only the hydrophic vegetation cover categories from the wet season map to use as an update feature, while the dry season map was used as an input feature. The new updated annual hydrophic indicator map will be combined with the other wetland indicator to produce a shore area wetland map saved as an output feature class. |
|  | 1.2 ii. How do you calculate vegetation indices, as you only mentioned SAR data? | We did not use vegetation indices for our classification purpose. Therefore we deleted from the manuscript, please check Attribute calculation sub-section. |
|  | **1.2 Wetland hydrology mapping using SAR data. Are the same SAR images used as for HV mapping? If so, how are permanent and temporary inundation identified, and was done by comparing these two months?**  **Line 258. “water bodies” is duplicated** | Please see Wetland hydrology mapping using SAR data sub-section-paragraph-2:  Permanent shore area wetlands, or those that are consistently inundated, are marked by a consistently low SAR backscatter in both the May and October SAR images. Our classification algorithm identifies these regions based on the stable low backscatter signature observed across both seasonal periods. Temporary/seasonal inundation of shore area wetlands category is characterized by a high change in backscatter between the two periods. We focus on areas that exhibit low backscatter in May, followed by a marked increase in backscatter in October. Finally, the annual wetland hydrology indicator map was produced by merging the two layers (temporary and permanently inundated layers produced from the dry and wet season images) using ArcGIS software: Data Management Tool of General: Merge.  Thank you, corrected. |
|  | **Reviewer-2 Comments** | **Response to Reviewer-2** |
|  | **Reviewer #2:** The authors addressed all the previous comments, and it is acceptable for publication from the scientific point of view. | Thank you. |
